# Multimorbidity and the inequalities of global ageing: a cross-sectional study of 28 countries using the World Health Surveys

**DOI:** 10.1186/s12889-015-2008-7

**Published:** 2015-08-13

**Authors:** Sara Afshar, Paul J. Roderick, Paul Kowal, Borislav D. Dimitrov, Allan G. Hill

**Affiliations:** Academic Unit of Primary Care and Population Sciences, Faculty of Medicine, University of Southampton, Southampton General Hospital, Tremona Road, Southampton, SO16 6YD UK; University of Newcastle Research Centre for Gender, Health and Ageing, Newcastle, NSW Australia; World Health Organization’s Study on global AGEing and adult health (SAGE), Geneva, Switzerland; Academic Unit of Social Statistics and Demography, Faculty of Social and Human Sciences, University of Southampton, Southampton, UK

**Keywords:** Multimorbidity, Ageing, Health inequalities, Epidemiological transition, Adult health

## Abstract

**Background:**

Multimorbidity defined as the “the coexistence of two or more chronic diseases” in one individual, is increasing in prevalence globally. The aim of this study is to compare the prevalence of multimorbidity across low and middle-income countries (LMICs), and to investigate patterns by age and education, as a proxy for socio-economic status (SES).

**Methods:**

Chronic disease data from 28 countries of the World Health Survey (2003) were extracted and inter-country socio-economic differences were examined by gross domestic product (GDP). Regression analyses were applied to examine associations of education with multimorbidity by region adjusted for age and sex distributions.

**Results:**

The mean world standardized multimorbidity prevalence for LMICs was 7.8 % (95 % CI, 7.79 % - 7.83 %). In all countries, multimorbidity increased significantly with age. A positive but non–linear relationship was found between country GDP and multimorbidity prevalence. Trend analyses of multimorbidity by education suggest that there are intergenerational differences, with a more inverse education gradient for younger adults compared to older adults. Higher education was significantly associated with a decreased risk of multimorbidity in the all-region analyses.

**Conclusions:**

Multimorbidity is a global phenomenon, not just affecting older adults in HICs. Policy makers worldwide need to address these health inequalities, and support the complex service needs of a growing multimorbid population.

**Electronic supplementary material:**

The online version of this article (doi:10.1186/s12889-015-2008-7) contains supplementary material, which is available to authorized users.

## Background

The theory of epidemiological transition is grounded on the observed shift of disease burden from communicable to non-communicable disease (NCD) causes [1]. Whilst the debate about the role of population ageing in epidemiological transition continues, the demographic transition to older populations is also occurring across all regions, albeit with different patterns, determinants and rapidity. It has been shown that the ageing of populations is ongoing in both developed and developing countries although, the growth rate of older adults in low- and middle-income countries will remain significantly higher than in most high-income countries (HICs) for many decades [2]. Multimorbidity is usually defined as the presence of two or more chronic diseases within an individual [3]. Although chronic disease factors are considered drivers of multimorbidity, the observed increase in multimorbidity is also related to both the demographic and epidemiologic transition. As the global population continues to grow in size, and becomes increasingly aged, there is an expectant increase in multimorbidity prevalence. Tackling multimorbidity as part of NCD burden remains one of the key challenges faced by the global community. In particular, health systems need to examine its socio-economic determinants in order to provide the most equitable health care to their populations and to drive NCD prevention.

Despite the growing recognition of the prevalence of multimorbidity amongst older adults, global prevalence studies have largely remained single-disease focused [4]. Few studies have reported national level estimates. Population prevalence studies in Spain and Germany suggest that multimorbidity prevalence is approximately 60 % for people aged 65 years and above [5, 6]. While the focus on older adults is common, multimorbidity also affects younger adults [7]. A study in Australia reported a multimorbidity prevalence of approximately 4 % in adults aged 20–39 years, 15 % in the 40–59 age group, and 39 % in those aged 60 and older [8]. There are also contrasting associations by age and sex. Multimorbidity in HICs is reportedly more prevalent for individuals of higher ages, female sex, low income, and low education [9–11]. The outcomes of multimorbidity have been well documented in HICs, with multimorbidity being associated with reduced quality of life, decreased functional capacity, and reduced survival [12–14]. Studies have also shown the burden of multimorbidity and its relation to rising healthcare utilisation, cost and expenditure [15, 16]. A comparison of the relationship between multimorbidity and socio-economic status (SES) show contrasting results for high, middle and low income countries. In Scotland, a high income country, multimorbidity has been found to be associated with lower SES [17]. In Bangladesh, a low income country - however, the wealthiest quintile of the population had an increased prevalence of multimorbidity [18]. And in studies examining its association with education, multimorbidity was more prevalent in those with lower educational levels in Canada (a HIC) [11]; while multimorbidity was less common among educated and employed persons in South Africa (an upper-middle income country) [19].

There have been no studies examining the age and socioeconomic distribution of multimorbidity (MM) in LMICs. The present study aims to establish the prevalence of MM in a range of LMICs, and to examine the variations of MM by age and education (as a proxy for SES).

## Methods

### Study samples

Publically available data from the WHO World Health Survey (WHS) was used, which is publicly available from the WHO. The World Health Surveys consists of cross-sectional national studies, each of which follow a multi-stage clustering design to draw nationally representative samples of adults aged 18 years and older. The details of the survey procedures are described elsewhere [20, 21]. Seventy-one countries participated in the WHS between 2001 and 2004. Sample sizes varied between countries depending on feasibility and cost. Individual participants aged 18 years or above were randomly selected for interview. All surveys were implemented as face-to-face interviews; except for two countries, which used phone and mail-in interviews.

Of the seventy-one countries that participated in the WHS, eighteen countries were excluded from the analyses, as they did not complete the long version of the questionnaire covering chronic condition status; these were mostly countries from Western Europe. Countries were also excluded if the response rate to the chronic health questions was less than 90 % (eleven countries) or if they did not include post-stratification weights (six countries). A minimum of four countries were randomly selected from each region for further analysis, resulting in a total of twenty-eight of the remaining thirty-seven countries. Since the research questions aimed to address the differences between LMICs the majority of countries sampled were LMICs. Due to low response rates in certain regions, such as Africa, countries from Eastern Europe & Central Asia were oversampled. We included one high income countries for comparison. In total, six countries were randomly selected from Africa; five countries from South-East Asia; four from South Asia; eight from Eastern Europe & Central Asia; four from Central & South America; and, one from Western Europe. Sampling weights were applied, as well as post-stratification weights to account for non-response.

### Measures and variables

In the WHS, chronic disease morbidity was defined by self-report, based on a set of six doctor diagnosed conditions. The self-reported conditions were assessed based on responses to the question, *“Have you ever been diagnosed with…?”* Previous studies have used different operational definitions of multimorbidity. Methodological differences, such as the number of chronic conditions to include in the count, result in a wide variability in prevalence estimates [7]. To prevent further discordance, multimorbidity is defined here as the presence of two or more chronic diseases, which is the most commonly used definition in prevalence studies [22]. A binary variable for multimorbidity was created on the presence of two or more of the six conditions: arthritis, angina or angina pectoris (a heart disease), asthma, depression, schizophrenia or psychosis, and diabetes.

The individual level socio-demographic variables of interest were age, sex and highest level of education completed. The residence of the individual, defined as living in either an ‘urban’ or ‘rural’ area, was also used in the description of the country characteristics. Two different age groupings were generated for different analyses: first, three age groupings for those 18–49 years, 50–64 years and 65+ years; and then by two groups for those younger than 55 (18–54 years) and those aged 55 years or older. The former was done to examine stratum specific differences, and the latter to examine generational differences. To examine generational differences, 55 years was taken as a cut point, representing a mid-way point within the WHS study population.

Level of education was used as a measure of country-level socioeconomic status (SES). *‘Highest education level obtained’* was collapsed from seven to four categories: (1) university or any higher education; (2) secondary school; (3) primary school; and, (4) less than primary school (including no formal education).

Inter-country socioeconomic differences were examined by using country estimates for GDP per capita. These were obtained from the United Nations Statistical Division records for 2003. Countries were then grouped according to the cut-offs for low- middle- and high-income based on the World Bank classification figures in 2003 [23].

### Statistical analysis

Survey estimates were used to calculate prevalence measures and extract nationally representative samples, accounting for non-response. To obtain valid comparisons across the countries, age-standardised multimorbidity prevalence rates were calculated using the direct method with the WHO Standard Population (2000–2025) [24]. For the descriptive analyses, mean percentages were taken as an average across populations and normality of the distributions was tested using the Shapiro-Wilk test. We used non-parametric regression to produce a line of best fit, when comparing national estimates of multimorbidity with GDP. Individual countries were weighted by the survey size to produce regional estimates for comparisons of multimorbidity by age and education. Significance testing of the comparisons among independent samples was done by t-test or ANOVA while for those whose distributions deviated from the normal one - by the Wilcoxon rank-sum (for two variables) and Kruskal-Wallis (for more than two variables) tests. ‘Prevalence ratios’ of multimorbidity by education were calculated with the reference category being primary school education completion. Univariable models were fitted to analyse the association of both sex and age with multimorbidity. For the multivariable analyses, data were pooled at regional level. A random effects logistic regression model was fitted for the regional analysis, to account for the hierarchical nature of the data within countries and regions. Odds ratios (OR) and 95 % confidence intervals (CI) are presented, with *p* < 0.05 taken as statistically significant, unless stated otherwise. All analyses were done using Stata version 12. Confidence intervals have been calculated based on recommendations for crude and age-specific rates [25].

## Results

Individual country characteristics are described in Table 1. Socio-demographic characteristics, including age and sex distributions are shown. Population age structures differed across the countries (*p* < 0.05), with a mean percentage of 9.0 % (95 % CI, 7.1 – 11.0) in those aged 65+ compared to 72.0 % (95 % CI, 68.4 – 75.7) in those aged 18–49. Age-standardisation of rates were calculated to account for these population distribution differences. The mean percentage of those living in rural areas was 49.2 % (95 % CI, 41.3 -57.1) compared to 50.8 % in urban areas (95 % CI, 42.9 -58.7), although the difference was not significant. Countries in Central Asia & Eastern Europe region had a higher proportion of individuals in the 65+ age category (mean = 14.6 %; 95 % CI, 12.5– 16.7) compared to the African region (mean = 5.3 %; 95 % CI, 4.1 – 6.4; *p* < 0.05).

**Table 1:** Sample size, age, sex and urban/rural distributions for the selected World Health Survey Countries

WHS Countries (n = 28)		N Sample	Age category, %			Sex, %	Residence, %	National income^a^
			18-49	50-64	65+	Female	Urban	
Africa	Burkina Faso	4948	82.8	12.7	4.5	52.8	17.8	LIC
	Ghana	4165	80.1	15.3	4.6	50.9	45.6	LIC
	Kenya	4640	87	9.6	3.4	51.2	39.9	LIC
	Morocco	5000	78.6	15.7	5.7	50.5	57.5	MIC
	Namibia	4379	78.5	13.4	8.1	53	33.2	MIC
	South Africa	2629	79.7	15	5.3	52	56.3	MIC
Central & South America	Brazil	5000	74.7	18.5	6.8	51.5	83	MIC
	Dominican Republic	5027	76.6	17	6.4	49.1	58.5	MIC
	Paraguay	5288	80	14.8	5.2	50.4	56.7	MIC
	Uruguay	2996	61.8	21.9	16.3	52.5	92.8	MIC
Central Asia & Eastern Europe	Bosnia & Herz	1031	66.4	21.6	12	51.1	44.6	MIC
	Czech Republic	949	57.8	27.4	14.8	52.1	73	MIC
	Estonia	1021	55.5	26.9	17.6	55.4	69.7	MIC
	Georgia	2950	60.9	23.8	15.3	53.3	51.5	MIC
	Hungary	1419	57.3	26.6	16.1	53.2	64.9	MIC
	Kazakhstan	4499	73.1	18.3	8.5	52.1	55.9	LIC
	Latvia	929	55.2	27.7	17.1	55.4	66.5	LIC
	Ukraine	2860	58.6	26.1	15.3	54.5	66.7	MIC
	Bangladesh	5942	81.1	14.7	4.2	48.5	24.3	LIC
South Asia	Mauritius	3968	73.6	18.7	7.7	50.8	43	LIC
	Pakistan	6502	76.4	19.2	4.4	49.6	33.9	MIC
	Sri Lanka	6805	71.5	20.6	7.9	47.9	20.6	MIC
	Laos	4989	80	15	5	50.7	20.3	LIC
South East Asia	Malaysia	6145	76.1	18.2	5.6	49.6	64.1	LIC
	Myanmar	6045	77	16.5	6.5	51.1	29.1	LIC
	Nepal	8822	78.1	16.8	5.1	49.5	15.2	MIC
	Philippines	10083	79.3	15.7	5.1	50.4	61.4	MIC
Western Europe	Spain	6373	59.2	22.1	18.7	51.5	76.8	HIC
	Mean	4478.7	72	18.9	9	51.5	50.8	

^a^ *MIC* Middle income country, *LIC* Low income country. All income groupings based on 2003 World Bank Estimates

Individual morbidity estimates suggest that arthritis is the most common condition across the WHS countries, with mean prevalence of 12.0 % (95 % CI, 11.8 - 12.2). The mean prevalence for depression, angina, asthma, diabetes and schizophrenia, respectively, were 6.7 %, 7.5 %, 5.0 %, 4.0 % and 0.9 % [see Additional file 1]. Multimorbidity prevalences by country are shown in Table 2. Both age-specific prevalences and age standardized prevalence are shown for each country. The mean world standardized prevalence for LMICs was 7.8 % (95 % CI, 6.5 – 9.1) and the range was 1.7 % (95 % CI, 1.4-2.0) to 15.2 % (14.3 – 16.0). The mean multimorbidity prevalence significantly increased with age in all countries (*p* < 0.05); 3.8 % (95 % CI, 3.0 – 4.6) for age 18–49, 12.8 % (95 % CI, 10.5 – 15.2) for 50–64; and 21.3 % (95 % CI, 17.1 – 25.5) for 65 + .

**Table 2:** Standardised multimorbidity prevalence by age category, with 2003 GDP per capita (in US$)

	Prevalence by age category (95 % CI)			Prevalence (95 % CI)^a^	GDP (US $)^b^
	18-49	50-64	65+		
Myanmar	1.30 (1.0 - 1.60 )	1.9 (1.0 - 2.7)	3.1 (1.7 – 4.5)	1.7 (1.4 – 2.0)	200.0
Nepal	10.1 (9.3 – 10.9)	24.8 (22.2 – 27.5)	30.2 (26.2 - 34.1)	15.2 (14.3 – 16.0)	264.0
Burkina Faso	4.8 (4.1 – 5.5)	9.7 (7.2 -12.2)	13.0 (9.0 - 16.9)	6.3 (5.6 – 7.0)	332.0
Laos	2.5 (2.0 – 3.0)	6.5 (4.6 – 8.4)	5.3 (2.7 – 7.8)	3.6 (3.1 – 4.1)	358.0
Bangladesh	2.9 (2.4 – 3.4)	10.9 (8.6 – 13.2)	12.6 (9.2 – 16.1)	6.8 (6.1- 7.5)	419.0
Kenya	2.1 (1.6- 2.5)	3.2 (1.8- 4.6)	11.5 (8.1 – 14.9)	4.2 (3.6 - 4.8)	440.0
Pakistan	3.4 (2.9 - 3.9)	8.7 (6.8 – 10.6)	14.8 (11.1- 18.5)	4.9 (4.3 – 5.4)	597.0
Ghana	2.0 (1.5 – 2.5)	4.4 (2.8 – 5.9)	6.6 (4.3 – 9.0)	3.6 (3.0 – 4.2)	603.0
Georgia	4.0 (3.0 - 5.1)	15.0 (11.8- 18.1)	27.1 (23.3 – 30.9)	9.6 (8.4 – 10.8)	874.0
Sri Lanka	1.2 (0.9 – 1.5)	6.6 (5.2- 8.1)	9.6 (7.1 - 12.0)	3.9 (3.4 – 4.3)	968.0
Philippines	3.8 (3.4 - 4.3)	12.0 (10.3 - 13.7)	17.2 (14.1 – 20.3)	7.1 (6.6 - 7.7)	1016.0
Ukraine	3.3 (2.4 - 4.2)	17.8 (14.6 – 20.9)	31.6 (27.1 – 36.1)	10.0 (8.8 – 11.1)	1049.0
Paraguay	3.2 (2.7 - 3.8)	9.4 (7.2 – 11.5)	12.0 (9.0 – 15.0)	5.7 (5.1 – 6.4)	1159.0
Morocco	3.0 (2.5 - 3.6)	13.6 (11.1- 16.1)	17.5 (13.8 - 21.1)	6.4 (5.7 – 7.1)	1684.0
Kazakhstan	1.5 (1.1 – 1.9)	10.1 (7.9 – 12.3)	45.1 (37.4 – 52.8)	8.5 (7.6 – 9.4)	2109.0
Bosnia & Herz	2.3 (1.0 – 3.5)	11.7 (7.3- 16.0)	30.2 (22.7 – 37.7)	7.6 (5.9 – 9.3)	2182.0
Dominican Republic	4.5 (3.7 - 5.2)	15.7 (13.0 – 18.5)	18.5 (14.9 – 22.1)	7.2 (6.4 – 8.0)	2210.0
Namibia	4.5 (3.7 - 5.2)	11.9 (8.9 – 14.9)	17.7 (13.4 – 21.9)	7.9 (7.0 - 8.8)	2489.0
Brazil	8.1 (7.1 – 9.0)	21.4 (18.4 - 24.4)	28.0 (23.7 – 32.3)	13.4 (12.4 - 14.5)	3039.0
South Africa	5.0 (3.9 – 6.0)	21.6 (16.6 – 26.6)	30.1 (20.6- 39.7)	11.2 (9.8 - 12.5)	3589.0
Uruguay	4.1 (3.2 - 5.0)	12.4 (9.7 – 15.1)	17.0 (13.5 - 20.5)	7.3 (6.3 – 8.2)	3622.0
Malaysia	2.0 (1.6 - 2.5)	9.6 (7.8 – 11.4)	14.6 (11.2 – 17.9)	5.6 (5.0- 6.2)	4607.0
Mauritius	3.3 (2.6 – 3.9)	15.8 (12.8 – 18.7)	19.3 (14.9 - 23.6)	7.8 (6.9 – 8.6)	4830.0
Latvia	2.7 (1.1 – 4.3)	16.0 (10.7 - 21.2)	35.6 (28.1- 43.0)	9.6 (7.5 – 11.7)	4872.0
Estonia	6.2 (4.0 – 8.4)	14.4 (9.9 – 18.8)	34.4 (26.8 – 41.9)	11.5 (9.4 - 13.6)	7350.0
Hungary	7.8 (5.8 – 9.9)	27.9 (22.5 – 33.3)	32.3 (26.2 – 38.3)	15.0 (13.0- 17.1)	8237.0
Czech Republic	3.5 (1.8 – 5.1)	11.6 (7.2 – 16.0)	39.4 (30.8 – 48.0)	9.4 (7.4 – 11.4)	9339.0
Spain	3.1 (2.5 – 3.8)	15.3 (13.3 – 17.3)	22.6 (20.5 – 24.6)	7.8 (7.1 - 8.5)	21035.0

World Mean Prevalence 7.8 (7.8-7.8)

^a^Multimorbidity prevalence ( ≥2 chronic conditions) standardised to the WHO Standard Population; ^b^National GDP per capita from the UN Division Statistical Division, 2003

**Fig. 1:**
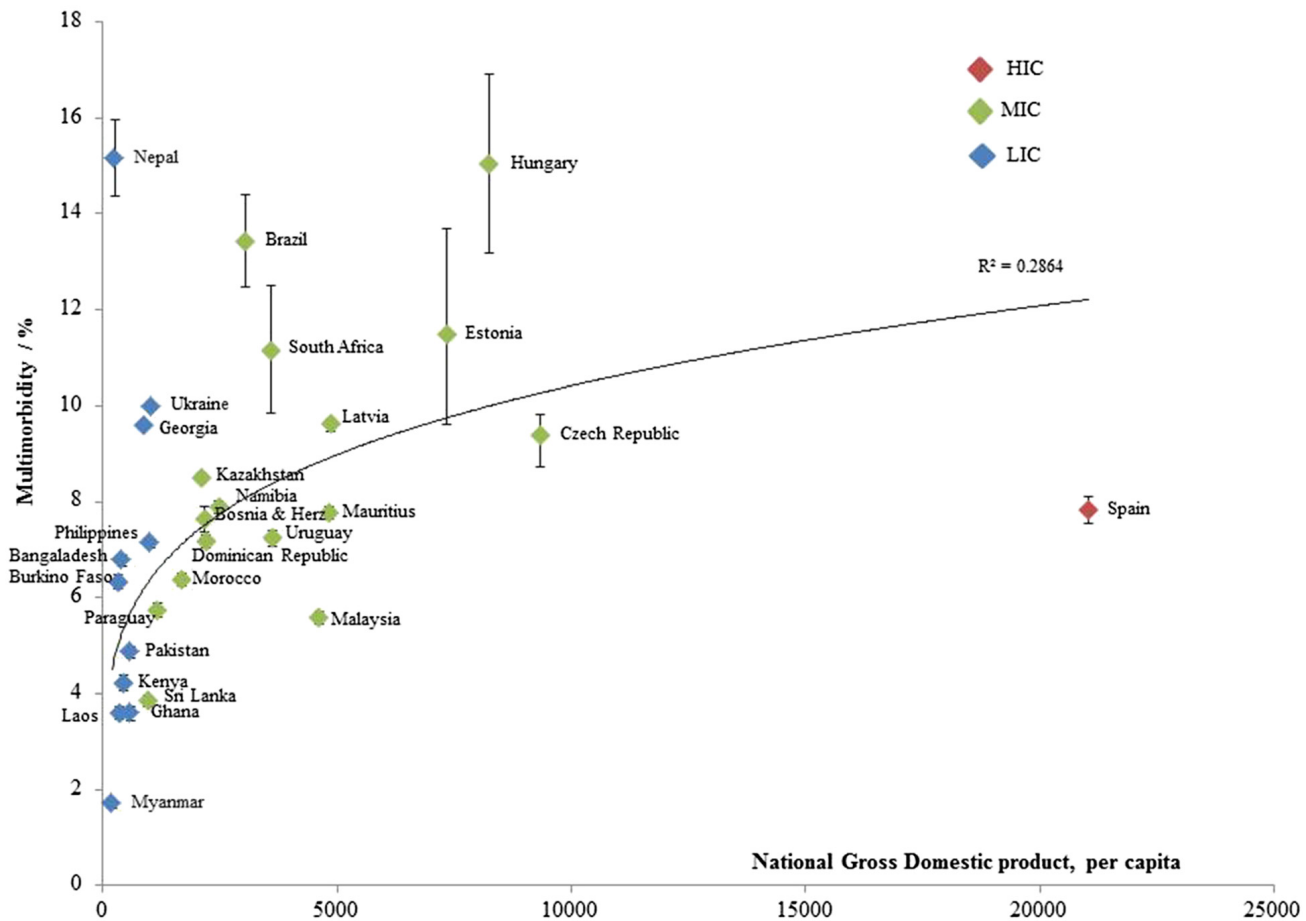
World Standardised Multimorbidity Prevalence for LMICs by GDP across World Health Survey Countries (*n* = 28) in 2003 (with confidence intervals). *HIC high income group; MIC middle income group; LIC low income group. Income groups are based on national estimates of 2001 GNI per capita, calculated using the World Bank Atlas method, and reported in the ‘World Development Report 2003’

Figure 1 shows national levels of multimorbidity by country GDP per capita. There was a positive association between multimorbidity prevalence and GDP per capita (from GDP per capita of $200 – $10,000). Above $10,000 the line flattens: Spain had a relatively low multimorbidity prevalence given their high GDP per capita.

**Fig. 2:**
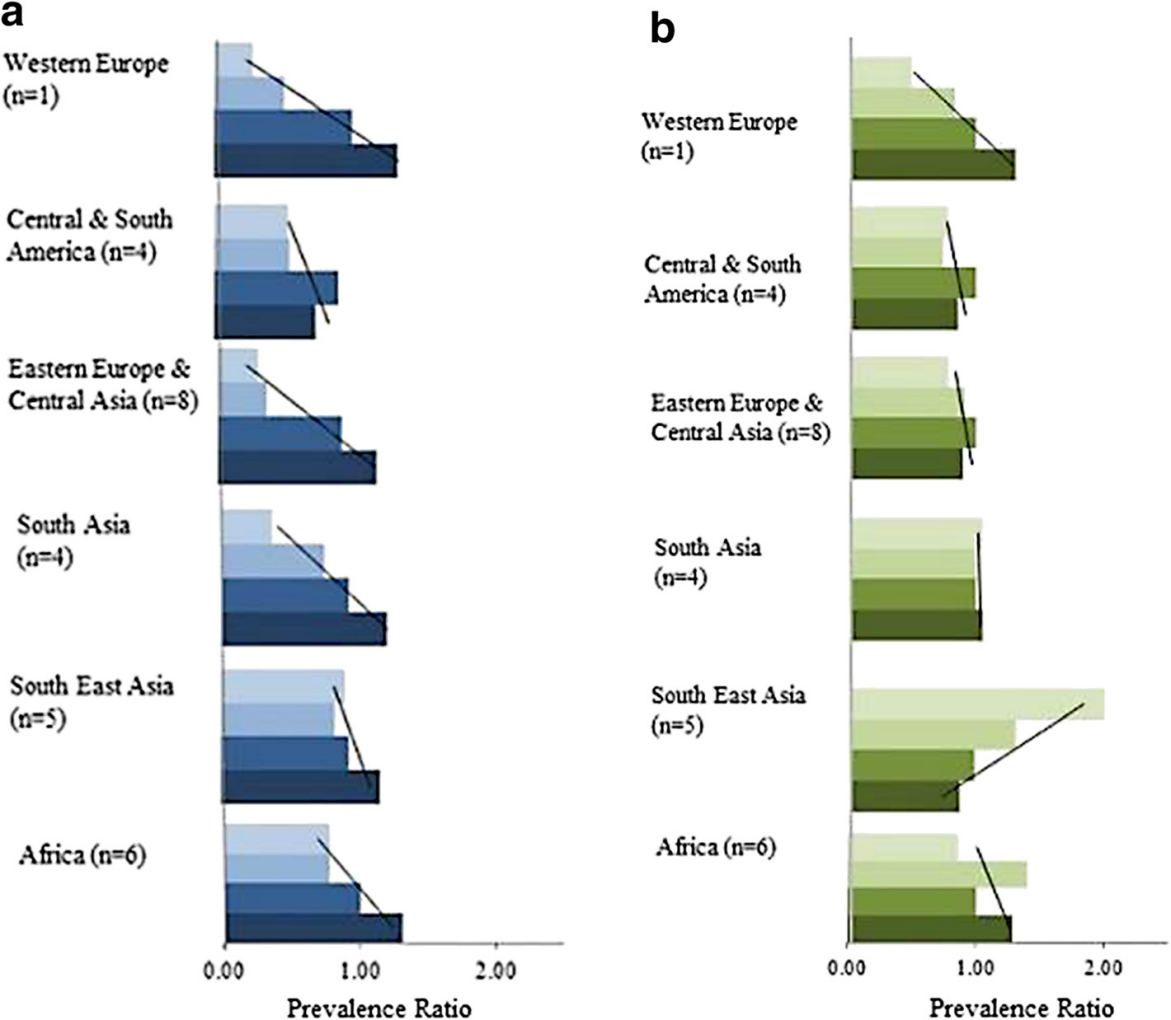
**a**: The socioeconomic gradient of multimorbidity by regions, for age category 1 (<55). **b**: The socioeconomic gradient of multimorbidity by regions, for age category 2 (≥55). The lightest shade represents the first category (higher education achieved). The darkest shade represents final category (less than primary school education achieved). Multimorbidity prevalence ratios are based on the prevalence of multimorbidity in the third category, set at 1

Figure 2 shows the prevalence ratios of multimorbidity across socioeconomic groups, stratified into younger and older adults. Amongst the younger adults, across all regions, there was a distinct negative socioeconomic gradient, with the highest burden on the least educated. In Western Europe there appeared to be a wider variation between SES categories, compared to SE Asia and Africa. Amongst older adults, there was less variation between SES categories, compared to the younger adults. However, there was still a distinct negative gradient in Western Europe, with the highest burden on the least educated. South-East Asia on the other hand has a positive gradient, with the highest burden on the most educated.

Both univariable and multivariable analyses are shown in Tables 3 and 4. Univariable and multivariable analyses at the country level are shown in Table 3, showing the sociodemographic correlates of age, sex and education. Age was significantly associated with multimorbidity in all countries. Sex was significantly associated with multimorbidity in all but seven countries. Multimorbidity was associated with education in the univariable analyses, but was not significant when adjusted for both age and sex, except for certain education categories in Bangladesh, Brazil, Hungary, Mauritius, Namibia and Spain; which were all consistent with an inverse relationship.

**Table 3:** Effect of age, sex and education on multimorbidity by country: Odds ratios in univariable, multivariable analysis

	Age (OR) 18–49 as reference		Sex (OR) Male as reference	Education (OR) primary school as reference			Education (AOR)		
	50 - 64	65+		< primary	Secondary	higher	< primary	secondary	higher
Burkina Faso	2.2*	3.0*	0.7***	1.1	0.5	1.1	0.9	0.5	1.1
Bangladesh	4.1*	4.9*	0.9	1.1	0.7	0.4***	0.8	0.7	0.4***
Bosnia & Herz	5.7*	18.7*	0.4***	3.0**	0.6	$	1.0	1.0	$
Brazil	3.1*	4.4*	0.5*	1.6*	0.6*	0.8	1.2	0.8***	0.8
Czech Republic	3.7*	18.1	0.6	2.3	0.3***	0.4	1.3	0.7	0.7
Dominican Republic	4.0*	4.8*	0.3*	2.0**	0.7	0.9	1.3	0.6	0.8
Estonia	2.6*	8.0*	0.6**	1.6	0.8	0.6	0.9	1.3	0.9
Georgia	4.2*	8.8*	0.6*	1.9	0.7	0.8	2.0	2.3	2.9
Ghana	2.2*	3.5*	0.6**	1.2	3.0	1.2	0.9	0.3	1.2
Hungary	4.6*	5.6*	0.5*	2.9	0.4*	0.2***	3.4***	0.8	0.5***
Kazakhstan	7.4*	54.2*	0.5*	0.3	0.2*	0.1***	0.5	1.1	0.8
Kenya	1.6*	6.2*	1.0	2.3*	1.7	2.5	1.4	1.8	2.4
Laos	2.7*	2.2*	0.9	1.5	0.5	0.2	1.3	0.5	0.3
Latvia	6.9*	20.0*	0.4**	1.6	0.9	1.0	0.7	1.7	1.3
Malaysia	5.2*	8.2*	0.8	1.8*	0.5*	0.5*	1.1	0.8	0.8
Mauritius	5.6*	7.1*	0.6*	2.8*	0.4*	0.4***	1.3	0.5*	0.4
Morocco	5.0*	6.8*	0.6***	1.4	0.5	0.4	0.6	0.6	0.7
Myanmar	1.4*	2.4*	0.5**	0.9	1.4	1.3	0.6	1.5	1.2
Namibia	2.7*	4.3*	0.6**	2.2*	0.7	1.2	1.7***	0.8	1.4
Nepal	2.9*	0.8*	1.0	1.3*	0.9	1.4	0.9	0.9	1.4
Pakistan	2.7*	4.9*	0.5**	1.4	0.6	1.1	0.9	0.6	1.1
Paraguay	3.0*	4.1*	0.3*	1.2	1.0	1.0	0.8	1.1	1.0
Philippines	3.4*	5.2*	0.6*	1.7*	0.8	1.1	1.3	1.1	1.3
South Africa	5.3*	8.3*	0.6*	2.4*	0.8	0.5	1.6	1.1	0.7
Spain	5.6*	9.1*	0.6*	1.8*	0.4*	0.2*	1.4***	0.8***	0.4**
Sri Lanka	5.9***	8.8***	0.7	1.2	0.5***	0.3	0.9	0.9	0.5
Ukraine	6.4***	13.7***	0.4***	0.7	0.5	0.3	0.6	1.4	1.3
Uruguay	3.3***	4.8***	0.5***	2.0**	0.8	0.7	1.4	1.0	0.9

*p-value *** < 0.05; ** < 0.01; * <0.001; (OR) Unadjusted odds ratio; (AOR) Adjusted odds ratios in multivariable analysis: all countries adjusted for age and sex. $ indicates no observations within the category. For Bosnia & Herzegovina the categories of secondary and higher education were combined for both univariable and multivariable analyses

**Table 4:** Effect of age, sex and socioeconomic status on multimorbidity: Odds ratios in univariable, multivariable analysis using a random effects model

	Univariable					Multivariable		
	Age (OR)	Sex (OR)	Education (OR)			Education (AOR)^$^		
	<55 years as reference	Male as reference	primary school as reference					
	≥55		<primary	secondary	higher	< primary	secondary	higher
Africa	3.3*	0.6*	1.8*	0.8	0.7	1.2**	0.9	0.8
Central & South America	3.0*	0.4*	1.5*	0.7*	0.8***	1.1	0.8***	0.8
Eastern Europe & Central Asia	6.0*	0.6*	1.4***	0.5*	0.5*	1.0	1.0	0.9
South Asia	4.1*	0.7 *	1.7*	0.6*	0.6**	1.2	0.7*	0.6**
South East Asia	3.3*	0.8*	1.4*	0.8*	0.9	1.1	0.9	1
Western Europe	6.0*	0.5*	1.6*	0.4*	0.2*	1.3**	0.7*	0.4*
All regions (MV adjusted for region)	3.7*	0.6*	1.5*	0.7*	0.6*	1.2*	0.9***	0.8*

*p*-value *** <0.05; ** < 0.01; * <0.001; ^$^Regional multivariable analyses adjusted for age, sex and country; (OR) Unadjusted odds ratio; (AOR) Adjusted odds ratios in multivariable analysis adjusted for age, sex and country

Similar to the country level, age and sex were both significantly associated with multimorbidity in all regions (Table 4). When adjusted for age and sex, the lowest education category was significantly associated with a higher risk of multimorbidity in Africa and Western Europe; and higher education categories were significantly associated with a decreased risk of multimorbidity in South Asia and Western Europe. Adjusted for age, sex, country and region, the ‘all region’ model suggests an overall negative education gradient.

## Discussion

The subject of multimorbidity is of growing interest, in part, due to the ageing of all populations. Internationally, there is still limited evidence on the prevalence and social determinants of multimorbidity, particularly in LMICs. This is the first study to describe global patterns of multimorbidity and to compare prevalence across different countries including LMICs. There are a few notable findings. Firstly, despite the variation in multimorbidity prevalence the mean world standard prevalence for LMICs was 7.8 % (95 % CI, 6.5 – 9.1), so even in LMICs the multimorbidity prevalence was quite high. Secondly, multimorbidity prevalence was positively associated with country GDP per capita. There was however a non-linear relationship; our one HIC - Spain had a low multimorbidity relative to per capita GDP. These results suggest an influence of other factors which may include, but are not limited to, more freedom to make better lifestyle choices and better social conditions [26]. In comparison to Spain, the Eastern European countries have relatively high multimorbidity prevalence. Historically, Eastern Europe has had poorer population health outcomes relative to their western counterparts following the fall of communism in 1990. Such health outcomes were markedly influenced by exposure to risk factors, such as tobacco smoking and alcohol consumption [27–29]. Thirdly, multimorbidity was significantly associated with age across all countries including LMICs. This finding has been found consistently across several studies [9, 17, 30–34]. Fourthly, multimorbidity as defined here, is also not limited to older adults, but affects younger adults in LMICs. This association of multimorbidity with age, however, might reflect the type of condition included in the disease count and their age of onset [35]. Fifthly, trend analyses of multimorbidity and education suggest a transgenerational difference: with a transition to a more negative education gradient is observed for younger adults compared to older adults in LMICs. Our ‘all region’ model also suggests an inverse relationship between multimorbidity and education. These findings are consistent with what has been found in other studies in HICs [17, 32]. Finally, there are notable gender differences in multimorbidity: the female sex being associated with higher multimorbidity. This is a common observation in morbidity studies, often attributed to greater use of health services and disease diagnosis [33]. Though other studies also suggest the role of other factors, including behavioural and psychosocial [34, 36]. Other studies suggest that clustering patterns of multimorbidity differ for male and females; for example, the cardiometabolic cluster was reportedly more common in males. This occurrence could be due to known differences in physiology, such as the protective effect of female hormones on CVD [37].

One of the study aims was to examine the variations of multimorbidity by SES here with education as a proxy. Our descriptive analyses of education show that both regional differences and generational differences exist for adults with multimorbidity. In Western Europe and Eastern Europe & Central Asia, there was wider variation in prevalence ratios between SES categories, compared to other regions. And for adults aged <55 years, the gradient was always negative, with one exception of older adults in South-East Asia. This suggests that in South-East Asia there might have been an inter-generational reversal in the socioeconomic gradient of multimorbidity. Such results have also been found in studies on obesity where transitional economies are experiencing a reversal in socioeconomic gradient thus resulting in a similar gradient to HICs [38].

The global-level multivariable analyses show a negative association of multimorbidity with education. Results from Western Europe (Spain) suggest a significantly negative education gradient of multimorbidity in HICs. In Africa, there is also a significantly negative education gradient in multimorbidity. The education gradient in Africa, despite most countries in this region being LMICs, is similar to the Western Europe region. These results are contrary to the Bangladesh study, which sampled 850 individuals (60 years and above) in a rural area and reported a direct association of multimorbidity with SES [18]. The SES index in their study, however, was based on household assets. Alternative measures of SES may lead to different results. One study in rural Uganda reports maternal education to be a better predictor of health; whereas other studies explore the use of permanent income [39, 40].

### Strengths and limitations

This study provides novel data on multimorbidity prevalence in nationally representative population samples using a consistent set of methods measures across multiple countries. Being the first of its kind, one of its major strengths is the availability and comparability of the data across all a wide range of countries using the World Health Surveys which were developed for this reason.

The study has few limitations which, even if not undermining its contributions and potential impact, should be also mentioned. Firstly, prevalence estimates were based on a limited set of conditions [7]. The chronic conditions included in the WHS were chosen to reflect health system coverage [41]. The conditions had to be amenable to self-report and reflect a known burden or prevalence globally. The choice of conditions should correspond to those with greater prevalence in older populations (prevalence for asthma, for instance, is more typically higher in older children and younger adulthood). Secondly, the study presents cross-sectional data from 2003. Further investigations should use current or recent data, as well as longitudinal data, to ascertain changing patterns over time. Thirdly, only countries with a greater than 90 % response rate to health status questions on chronic disease were sampled, which meant that a number of lower income countries, where response rates were low, were excluded from the analyses. There was also low representation from HICs, as these countries largely did not complete the chronic disease questions. As such the use of Spain only – to represent Western Europe – was a limitation. Fourthly, these results were based on self-reported measures, which may result in disease underreporting and potential bias [42–44]. One study notes that self-reporting leads to underreporting, particularly amongst the poor, which dampen the gradients [45]. It may be that health literacy and service access impact prevalence based on self-report for countries at different levels of economic development. Self-reported diagnosis can be further validated by auxiliary symptom-reporting questions included in the survey, such as the Rose questionnaire used for angina, or through clinical assessment [46]. National GDP is generally correlated with healthcare system investment and potentially healthcare access, which might affect the interpretation of the results. Spain, however, had low multimorbidity relative to national GDP despite having a relatively good healthcare system access. In order to understand the relationship between a country’s development and multimorbidity as an appropriate health outcome, further studies are needed: with a fuller accounting of confounding, modifying and mediating elements. Finally the use of education as a proxy for SES has been debated despite its wide use in population health research [47, 48]. There is evidence to suggest that after conditioning for the effect of socio-economic status, measured by household income or assets, education has an independent and substantial effect on health outcomes [49].

## Conclusion

Multimorbidity is common in LMICs and significantly associated with age. There is an inverse country association of multimorbidity with education, which indicates an inequity of disease burden. The negative gradient of multimorbidity with education is already occurring and more marked in the younger generation. It may reflect the proliferation of several key risk factors for these chronic conditions including unhealthy behaviours. The recent UN World Summit addressed the common risk factors of NCDs to be tackled with urgent priority; namely tobacco use, unhealthy diet, harmful use of alcohol and physical inactivity [50]. Weak health systems and governance will not be able to support the care needs resulting from the complexities of a multimorbid population. Better coordination and support through informed policy and planning of health care systems is needed to support the transition required for health systems to address future care needs. Furthermore, there is a need to increase activities and expand measures to reduce the modifiable risk factors that are driving multimorbidity prevalence.

## Additional file

Additional file 1:
**Shows the crude morbidity prevalence of chronic disease by country and region.**
