# Supplementary material for: Multimorbidity and the inequalities of global ageing: a cross-sectional study of 28 countries using the World Health Surveys
Source: BMC Public Health. 2015 Aug 13;15:776. doi: 10.1186/s12889-015-2008-7 (PMC4534141; doi:10.1186/s12889-015-2008-7)
Supplement: Additional file 1: — Shows the crude morbidity prevalence of chronic disease by country and region. [file 12889_2015_2008_MOESM1_ESM.docx]

Additional File 1

|  |  | **Crude Morbidity Prevalence, %** | | | | | |
| --- | --- | --- | --- | --- | --- | --- | --- |
|  |  | Angina | Arthritis | Asthma | Diabetes | Depression | Schizophrenia |
| **AFRICA** | Burkina Faso | 11.8 | 12.7 | 2.4 | 0.5 | 2.6 | 1.1 |
|  | Ghana | 4.6 | 7.0 | 4.2 | 0.9 | 1.5 | 0.7 |
|  | Kenya | 2.5 | 4.1 | 2.9 | 1.3 | 5.5 | 0.7 |
|  | Namibia | 7.7 | 10.1 | 3.6 | 2.1 | 7.7 | 3.0 |
|  | Morocco | 5.1 | 17.2 | 3.4 | 3.9 | 3.0 | 0.7 |
|  | South Africa | 4.7 | 10.0 | 6.3 | 8.6 | 9.0 | 1.2 |
| **CENTRAL & SOUTH AMERICA** | Paraguay | 5.4 | 3.8 | 5.9 | 4.2 | 6.7 | 0.5 |
|  | Uruguay | 5.7 | 9.5 | 8.7 | 5.1 | 10.5 | 0.7 |
|  | Dominican Republic | 3.8 | 11.3 | 9.6 | 4.0 | 8.5 | 0.9 |
|  | Brazil | 6.2 | 9.6 | 12.1 | 5.5 | 18.9 | 1.6 |
| **EASTERN EUROPE & CENTRAL ASIA** | Kazakhstan | 11.8 | 15.2 | 1.8 | 1.9 | 1.6 | 0.5 |
|  | Bosnia & Herz | 8.0 | 11.9 | 3.5 | 4.9 | 6.4 | 0.1 |
|  | Czech Republic | 6.4 | 19.1 | 4.7 | 10.4 | 5.8 | 0.5 |
|  | Estonia | 16.0 | 21.0 | 4.7 | 3.8 | 8.8 | 1.4 |
|  | Latvia | 18.9 | 13.2 | 4.2 | 5.5 | 5.9 | 0.7 |
|  | Ukraine | 18.9 | 17.1 | 4.4 | 3.1 | 3.7 | 0.7 |
|  | Georgia | 13.6 | 17.5 | 3.8 | 2.6 | 5.4 | 0.5 |
|  | Hungary | 16.6 | 25.4 | 6.8 | 8.8 | 8.4 | 2.4 |
| **SOUTH ASIA** | Bangladesh | 6.7 | 10.8 | 4.4 | 2.5 | 1.3 | 0.7 |
|  | Pakistan | 3.1 | 13.4 | 4.1 | 3.1 | 2.6 | 1.1 |
|  | Sri Lanka | 2.9 | 6.3 | 3.8 | 2.7 | 1.1 | 0.7 |
|  | Mauritius | 4.2 | 7.3 | 4.6 | 9.1 | 6.3 | 0.6 |
| **SOUTH EAST ASIA** | Myanmar | 2.7 | 4.3 | 2.9 | 0.5 | 0.5 | 0.3 |
|  | Nepal | 5.5 | 14.1 | 3.9 | 2.7 | 33.6 | 2.6 |
|  | Laos | 4.7 | 8.0 | 3.4 | 0.5 | 1.9 | 0.4 |
|  | Philippines | 5.6 | 12.7 | 8.0 | 2.1 | 3.7 | 0.4 |
|  | Malaysia | 3.4 | 8.7 | 5.9 | 5.0 | 2.6 | 0.2 |
| **WESTERN EUROPE** | Spain | 3.8 | 13.8 | 7.2 | 6.7 | 13.8 | 0.5 |
|  |  | *7.5* | *12.0* | *5.0* | *4.0* | *6.7* | *0.9* |
|  | *Mean (n=28)* |  |  |  |  |  |  |
